# Effectiveness of the intelligent hypertension excellence centers (iHEC) therapy model in the blood pressure management of older hypertensive patients: a randomized controlled trial

**DOI:** 10.1038/s41440-024-01951-w

**Published:** 2024-11-06

**Authors:** Yuqin Jiang, Lijun Zheng, Yunhe Zhang, Zhen Fan, Jianshu Guo, Junling Yang, Peng Liu, Ping Zhong, Dili Xie

**Affiliations:** 1 Medical School of University of Electronic Science and Technology of China, Chengdu, China; 2 Department of Geriatric Cardiology, Sichuan Provincial People’s Hospital, school of Medicine, University of Electronic Science and Technology of China, Chengdu, China; 3 Department of Medical Library, Sichuan Provincial People’s Hospital, school of Medicine, University of Electronic Science and Technology of China, Chengdu, China

**Keywords:** hypertension, intelligent, blood pressure management, older

## Abstract

Intelligent hypertension excellence centers (iHEC) may improve blood pressure (BP) management in older hypertensive patients. However, this has not yet been rigorously evaluated, so we conducted a prospective randomized open-label clinical trial to verify this hypothesis. Older patients with hypertension were recruited between January and June 2022. The control group received conventional treatment and visited doctors in clinic. The intervention group received the iHEC therapy model, including remote BP management, online consultation, and follow-up services with support from the internet. Both groups received 12 months follow-up. Finally, 540 older patients with hypertension participated in the study; of these, 517 completed the follow-up. The average age was 71.4 ± 3.7years, 81 patients with frailty (15.7%). When follow-up was terminated, the SBP of the intervention group was 4.2 mmHg lower than that of the control group (95% CI, 2.0 to 6.4, *P* < 0.001), and the overall BP control rate in the intervention group was higher than that in the control group (60.2% vs. 48.1%, *P* < 0.05). During follow-up, the new-onset rate of excessive BP lowering in the intervention group was lower than that in the control group (3.8% vs. 9.0%, *P* < 0.05). Patients with a median age or above and high school education or above had higher numbers of online consultations and home BP measurements (*P* < 0.05).Our study confirmed those who received the iHEC therapy model achieved better BP reduction, higher rates of BP control, and alleviated the risk of excessive BP reduction.

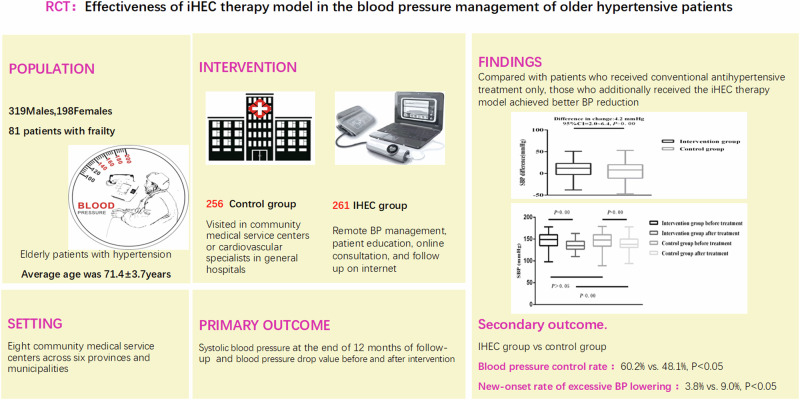

## Introduction

Hypertension is one of the most common chronic diseases worldwide, and is the primary risk factor for CVD [1, 2]. In China, the total number of patients with hypertension has reached 350 million, and more than half of them are older. However, only in approximately 15% of these patients have achieved blood pressure (BP) control targets [3]. Personal and social factors lead to poor hypertension management in older patients, such as age-related declines in cognitive function and memory, inconveniences of going out, difficulties in outpatient registration, and the high costs of visiting doctors. These unfavorable factors result in low compliance and losing of follow-up. Improving this situation would require new ideas.

Management of blood pressure in older patients has several unique characteristics. Systolic blood pressure (SBP) is strongly associated with target organ damage and cardiovascular events [4, 5]. Post-hoc analysis of the STEP study also demonstrated that the benefits of antihypertensive treatment in the older mainly depend on a reduction in SBP [6]. Therefore, more attention should be paid to the SBP in older patients. However, some older patients experience excessive SBP reduction during treatment, that leading to repeated incidents of dizziness, falls, and even fracture [7, 8]. The SBP should be maintained within a reasonable range, particularly in older patients with frailty. Therefore, it is important to avoid excessive SBP reduction.

With the rapid development of the Internet and the widespread adoption of smartphones, APP are becoming a new tool for healthcare [9, 10]. Mobile health(mHealth) is a new solution to chronic disease management [11, 12]. Initial mHealth studies on hypertension have been conducted in Europe and America and have gradually expanded to developing countries in recent years [13–15]. However, the effectiveness of the APP-based management remains debatable [16, 17]. The hypertensive patients included in previous studies on mHealth were relatively younger [18, 19], considering that operating smartphones may be a new challenge for the older. Therefore, whether this model is applicable to older patients remains unknown.

Intelligent hypertension excellence centers (iHEC) are a new model for hypertension management, promoted by the China Hypertension Alliance. The iHEC-APP, developed by Shanghai Zhizhong Medical Technology Co., Ltd., aims to build a nationwide professional technical platform for hypertension management, and connecting secondary and tertiary hospitals with community health service centers to achieve collaborative management of hypertension. This study aimed to clarify whether this model can improve the management of hypertension in older patients.

Point of view

- Clinical relevance
  IHEC program improved blood pressure control and solved the inconvenience of medical visits for older hypertensive patients, that worthy of clinical promotion.
- Future direction
  Construct regionalized intelligent management system for hypertension based on the iHEC platform throughout the country.
- Consideration for the Asian population
  The experience from this study can adapted to improve blood pressure management among other Asian population.

## Methods

### Study design

This prospective, non-blinded, randomized controlled clinical trial was supported by Sichuan Science and Technology Program (grant number: 2022YFS0355) and was approved by the Institutional Review Committee of Sichuan Provincial People’s Hospital (Ethical Review No.230 2022). The study adhered to the ethical standards of the 2008 Declaration of Helsinki. All the participants provided written informed consent. The participants were recruited from January to June 2022 and assigned randomly to the intervention group (receiving conventional treatment and iHEC therapy) and the control group (receiving conventional treatment only), with follow-up from July 1, 2022, to June 30, 2023. The main outcome was SBP at the end of 12 months of follow-up, adjusted for baseline SBP and sex.

### Participants

This study recruited older patients with hypertension from community medical service centers affiliated with general hospitals in various provinces and municipalities of China. The inclusion and exclusion criteria are presented in Table 1.

**Table 1:** Inclusion and exclusion criteria for study subjects

Inclusion criteria:	Exclusion criteria:
• Patients diagnosed with hypertension according to the “2019 Chinese guideline for the management of hypertension in the elderly”[20], including patients receiving or not receiving antihypertensive treatment • Aged between 60 to 80 years old. • Using smartphones by themselves (Android smartphone or iPhone) • Not included in other internet-based hypertension management programs • Permanent residents in the community • Agreement to participate and willingness to cooperate with the study	• Patients with refractory hypertension or currently taking more than 4 types of antihypertensive medications • SBP ≥ 180 mmHg or DBP ≥ 110 mmHg. • Orthostatic hypotension, white-coat hypertension, difference in BP between two upper limbs ≥20 mmHg. • Acute coronary syndrome, acute decompensated heart failure, stroke, or other severe cardiovascular and cerebrovascular events in the past six months. • Severe complications, such as CKD 4-5 stage, chronic heart failure with NYHA class III-IV, LVEF < 40%, or other serious systemic organic diseases. • Unable to cooperate due to physiological, psychological, cognitive impairment, or other reasons.

### Recruitment and randomization

General practitioners at community medical service centers identified potentially eligible participants based on their BP and clinical data. Patients diagnosed with hypertension according to the “2019 Chinese guideline for the management of hypertension in the elderly”: for patients not receiving treatment, measure office blood pressure three times on different days, with SBP ≥ 140 and/or DBP ≥ 90 mmHg, and those who have been clearly diagnosed with hypertension and are undergoing antihypertensive drug treatment at present.

We introduced the content of this study to potential participants using leaflets. One week later, patients were asked about their willingness to participate. Upon agreement, they were notified by text messages to participate in the study and asked to personally confirm their eligibility and receive guidance. After recruitment, a list of participants was entered into the random database. The participants were randomly allocated to the control and intervention group using SAS9.4 software with a 1:1 ratio. A non-deterministic minimal algorithm was used to ensure a balance in age, sex, baseline BP, smoking, and drinking habits between the two groups. None of the medical staff members were aware of the allocation sequences.

General practitioners guided all participants to measure BP following the “ 2019 Chinese guideline for the management of hypertension in the elderly” [20]. The study provided calibrated Omron BP73A3T upper-arm electronic BP monitors to all participants for free, both the intervention and control group. The participants were required to be demonstrated on-site to ensure correct operation.

The baseline data included age, sex, body mass index (BMI), disease duration, social history (smoking, alcohol consumption, and education level), baseline BP, and antihypertensive medication use. Smoking was defined as smoking more than once a day continuously or cumulatively for six months. Alcohol consumption was defined as drinking alcohol at least twice a month during the previous year. The FRAIL frailty score was used to assess the frailty status, with a score of 0 indicating no frailty, 1-2 indicating pre-frailty, and ≥3 indicating frailty. Mini-Cog scale was used for screening and identification of cognitive function, participants with Mini-Cog≤2 was considered cognitive impairment.

### Interventions

The control group received conventional treatment. Patients visited general practitioners in community medical service centers or cardiovascular specialists in general hospitals. The doctors provided antihypertensive treatment and outpatient follow-up, referring to the “ 2019 Chinese guideline for the management of hypertension in the elderly” [20]: long-acting antihypertensive, such as CCBs, ACEI/ARB/ARNI, diuretics, were used for the initiation or maintenance therapy. For most patients with an initial blood pressure above the target value by 20 mmHg or more, or those whose blood pressure is not controlled with monotherapy, combination therapy with CCBs + ACEI/ARB, ACEI/ARB+ diuretic can be considered. This may involve a fixed-dose combination or a free combination approach. If blood pressure control remains suboptimal, further addition of thiazide-like diuretics for blood pressure reduction may be warranted.

**Fig. 1:**
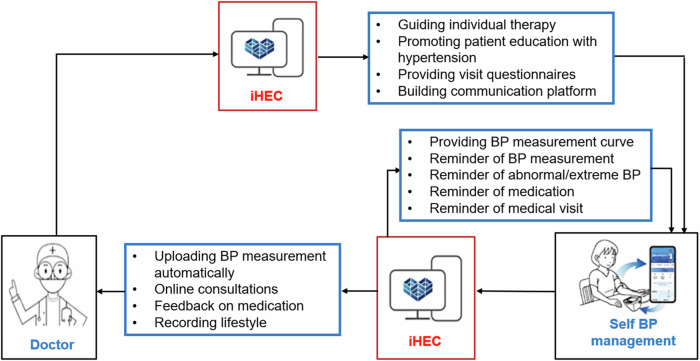
The process of the iHEC Therapy Model

The intervention group received iHEC for BP management and conventional treatment. Both patients and medical staff downloaded the iHEC-APP to their smartphones. This APP is an intelligent BP management tool that include iHEC health assistant mini-program for patients, iHEC-Web medical workstation for healthcare professionals, and intelligent BP official accounts to promote education. It should be emphasized that the Bluetooth connection of the smartphone should be turned on to ensure that the BP monitors synchronize with the patient’s smartphone and automatically upload real-time BP measurement data. The iHEC therapy model is shown in Fig. 1.

iHEC Health Assistant Mini-Program: We distributed detailed operating instructions of this mini-program to the participants. The research assistants guided the participants to download and install the iHEC-APP and then repeatedly demonstrated various functions, including how to receive and view reminders of BP measurement, abnormal BP, extreme BP, medication taking and medical visit, BP control curve, and how to apply for online consultation. This study focused on the management of abnormal and extreme BP. All patients were familiar with this APP within 1-2 weeks, then they were required to operate on-site independently. If patients felt confused about the APP, they could contact the research assistants and seek help.

iHEC-Web medical workstation: General practitioners in community medical service centers and the corresponding cardiovascular specialists in general hospitals participated jointly in remote personalized BP management for patients, including receiving BP data, conducting online consultations, and resource sharing.

Intelligent BP official account: Regularly pushed education on hypertension (involving the recommended lifestyle, how to improve treatment compliance, knowledge of antihypertensive medications, Cardiovascular risk factor screening, etc.), provided follow-up questionnaires, and built patient-communication platforms.

### Measurements and outcomes

At baseline and at the end of follow-up, all participants were required to measure their BP in the office using calibrated Omron electronic upper-arm BP monitors. SBP and DBP at baseline were recorded as the average of three measurements taken on separate days within the week prior to randomization. Similarly, SBP and DBP at the end of follow-up were recorded as the average of three measurements taken on separate days within the week after the end of follow-up.

The main Outcome was SBP at the end of 12 months of follow-up. Secondary Outcomes included the BP control rate (BP controlled within 140/90 mmHg was considered to meet the target, according to the “ 2019 Chinese guideline for the management of hypertension in the elderly” [20] and “2020 International Society of Hypertension Global Hypertension Practice Guidelines”[21]), cases of excessive BP reduction (referring to the STEP study [22] which focused on hypertension management strategies in older Chinese patients, SBP < 110 mmHg (more than three times) was considered as a criterion of excessive BP reduction, including patients receiving antihypertensive treatment with SBP < 110 mmHg at baseline, and patients with new-onset SBP < 110 mmHg during the follow-up), and the use of the iHEC APP in the intervention group.

The iHEC-APP usage data for the intervention group were extracted from Shanghai Zhizhong Medical Technology Co. Ltd.

### Sample size and power analysis

Previous studies [23, 24] have indicated that an SBP decrease of 5 mmHg can reduce coronary heart disease and stroke; therefore, the clinically significant SBP reduction in this study was set at 5 mmHg. Setting α = 0.05 (two-sided), the combined standard deviation of SBP was set at 16 mmHg for both groups, considering a 20% dropout rate (which might actually be lower). A minimum of 270 patients per group were required to detect a significant difference in SBP between the intervention and control groups. A total of 540 patients were enrolled in this study.

### Statistical methods

All analyses used intention-to-treat principles and followed a previously described protocol. The main outcome analysis was set as SBP at the end of follow-up and was analyzed using a linear regression model, correcting for sex and baseline SBP. Subgroup analyses were conducted using linear regression models to analyze specific effect factors (sex, BMI, disease duration, education level, frailty, and baseline SBP control) and to test for interactions to clarify whether the intervention effects were consistent across subgroups. All statistical tests were two-sided, with *P* < 0.05 indicating statistical significance. SAS software version 9.3 (SAS Institute Cary NC) was used for the statistical analyses.

## Results

### Participants

**Fig. 2:**
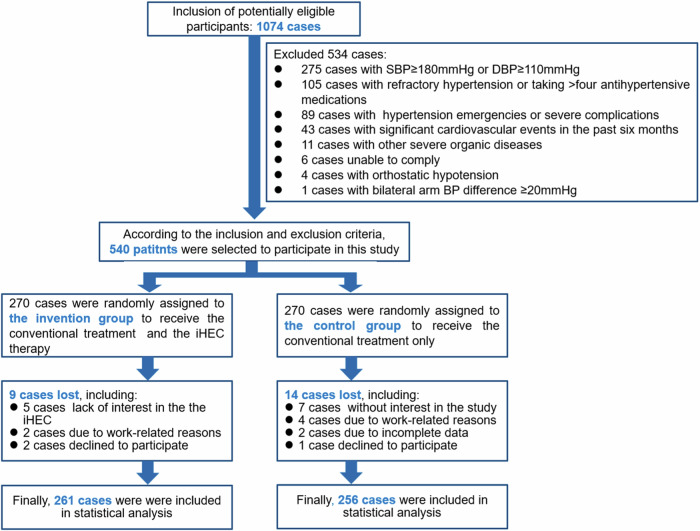
Flowchart of iHEC therapy model (Recruitment, Randomization, and Participant)

A total of 1074 potentially eligible participants were identified from eight community medical service centers across six provinces and municipalities in China. Among them, 540 patients who met the inclusion criteria were randomly assigned to either the intervention (270 patients) or the control (270 patients) group. All patients were able to upload their BP data correctly during follow-up. In total, 519 patients completed the follow-up period (261 in the intervention group and 258 in the control group). However, owing to storage errors, the BP data of two patients in the control group were missing; therefore, they were excluded. Finally, the data of 517 patients (95.7%) were used to analyze the results (261 patients in the intervention vs. 256 patients in the control group, respectively). A flowchart of the iHEC therapy model is shown in Fig. 2.

### Baseline characteristics

The average age of the participants was 71.4 ± 3.7years, with 198 female participants (38.3%). In both groups, the majority of patients were of the Han ethnicity (intervention group: 225 patients (86.2%); control group: 227 patients (88.7%)). The levels of education were similar in the two groups (those with high school education or above: intervention group, 125 patients (47.9%); control group, 117 patients (45.7%)). The frailty rates were also similar (intervention group, 40 patients (15.3%); control group, 41 patients (16.0%)). There were no statistically significant differences in BMI, marital status, smoking and drinking habits, types of antihypertensive drugs used, or comorbidities between the intervention and control groups, and the intervention and control groups were comparable. Table 2.

**Table 2:** Comparison of baseline characteristics between the two groups

Item		Intervention group (*n* = 261)	Control group (*n* = 256)	Statistical values	*P* values
Gender (*n*,%)	Male Female	157 (60.2) 104 (39.8)	162 (63.3) 94 (36.7)	0.54	0.46
Nationality (*n*,%)	Han ethnicity Minority nationality	225 (86.2) 36 (13.8)	227 (88.7) 29 (11.3)	0.71	0.40
Age (years),mean (SD)		71.6 ± 3.7	71.1 ± 3.8	1.26	0.21
BMI (kg/m^2^),mean (SD)		24.5 ± 3.3	24.00 ± 3.1	1.62	0.11
Education (*n*,%)	Junior school and below	136 (52.1)	139 (54.3)	0.25	0.62
	Senior school and above	125 (47.9)	117 (45.7)		
Marital status (*n*,%)	Married	191 (73.2)	189 (73.8)	0.03	0.87
	Unmarried/divorced/widowed	70 (26.8)	67 (26.2)		
Current smoking (*n*,%)		29 (11.1)	31 (12.1)	0.13	0.73
Current alcohol consumption (*n*,%)		54 (20.7)	47 (18.4)	0.45	0.50
Frailty (*n*,%)		40 (15.3)	41 (16.0)	0.05	0.83
Types of antihypertensive medication used (*n*,%)	0	82 (31.4)	79 (30.9)	0.26	0.97
	1	66 (25.3)	64 (25.0)		
	2	88 (33.7)	5 (33.2)		
	3	25 (9.6)	28 (10.9)		
Comorbidity (*n*,%)	Hyperlipidemia	44 (16.9)	41 (16.0)	0.07	0.80
	Diabetes	62 (23.8)	67 (26.2)	0.40	0.53
	Asthma/ COPD	49 (18.8)	45 (17.6)	0.12	0.72
	Coronary heart disease	9 (3.5)	8 (3.1)	0.04	0.84
	Stroke	11 (4.2)	10 (3.9)	0.03	0.86
	Heart failure	3 (1.2)	4 (1.6)	0.17	0.68
	AF	6 (2.3)	3 (1.6)	0.37	0.54

*BMI* body mass index, *COPD* chronic obstructive pulmonary disease, *AF* atrial fibrillation, *SD* standard deviation

### Blood pressure

At baseline, the SBP of the intervention and control groups were 149 (135, 160)mmHg and 148 (134, 160)mmHg, respectively. At the end of follow-up, SBP in the intervention group decreased by 12 (−3, 23)mmHg compared with baseline, whereas SBP in the control group decreased by 8 (−11, 20)mmHg compared with baseline. With the between-group adjustment, the SBP in the intervention group was 4.2 mmHg lower than that in the control group (95% CI, 2.0 mmHg to 6.4 mmHg, *P* < 0.001).

At baseline, the DBP of the intervention and control groups was 69 (63,78) mmHg and 70 (64,78) mmHg, respectively. At the end of follow-up, DBP in the intervention group decreased by 7 (−2,14) mmHg compared to baseline, while DBP in the control group decreased by 6 (−3,13) mmHg compared to baseline. With the between-group adjustment, DBP in the intervention group was 1.4 mmHg lower than the control group (95% CI, −0.1 mmHg to 2.9 mmHg), but the difference was not statistically significant (*P* > 0.05). Table 3.

**Table 3:** Comparison of blood pressure drop value before and after intervention in the two groups

	Intervention group (*n* = 261)			Control group (*n* = 256)			Corrected difference (95%CI)	*P* values
	Baseline	Month 12	Change	Baseline	Month 12	Change		
SBP(mmHg) median, (IQR)	149 (135,160)	135 (127,145)	12 (−3,23)	148 (134,160)	138 (131,150)	8 (−11,20)	4.2 (2.0,6.4)	＜0.01
DBP(mmHg) median, (IQR)	69 (63,78)	63 (58,69)	7 (−2,14)	70 (64,78)	64 (59,69)	6 (−3,13)	1.1 (−0.1,2.9)	0.09

*SBP* systolic blood pressure, *DBP* diastolic blood pressure, *IQR* interquartile range, *CI* confidence internal

We further analyzed the differences in the primary outcome across subgroups (sex, BMI, disease duration, education level, frailty, and baseline SBP control) between the two groups. The results showed no statistically significant heterogeneity in the intervention effect across these subgroups (*P*-values for intervention-by-subgroup interaction terms were>0.05). Table 4 and Supplementary Fig. 1.

**Table 4:** Subgroup analysis of differences in systolic blood pressure(mmHg) before and after intervention in the two groups

Item		Intervention group (*n* = 261)			Control group (*n* = 256)			Corrected difference (95%CI)	*P* values
		*N*	Baseline median (IQR)	Month 12 median (IQR)	*n*	Baseline median (IQR)	Month 12 median (IQR)		
Gender	Male	157	149 (136,160)	135 (127,144)	162	148 (134,160)	137 (132,150)	4.0 (1.3,6.7)	0.99
	Female	104	150 (134,160)	135 (128,147)	94	148 (134,159)	139 (131,150)	3.8 (0.4,7.2)	
Nationality	Han ethnicity	225	149 (135,160)	135 (127,145)	227	148 (133,160)	138 (131,150)	3.5 (1.3,5.7)	0.41
	Minority nationality	36	150 (135,162)	135 (123,150)	29	150 (134,161)	139 (135,151)	6.1 (0.1,12.3)	
BMI	<24	125	148 (133,158)	135 (127,145)	122	148 (133,160)	138 (130,151)	3.9 (0.5,7.3)	0.97
	≥24	136	150 (136,162)	135 (128,146)	134	148 (136,160)	137 (132,150)	3.9 (1.3,6.5)	
Education	Junior school and below	136	149 (135,162)	134 (128,143)	139	148 (134,160)	138 (132,150)	4.5 (1.7,7.4)	0.52
	Senior school and above	125	149 (134,159)	135 (127,146)	117	148 (134,159)	137 (131,151)	3.1 (0.0,6.3)	
Frailty	Yes	40	148 (138,158)	134 (123,137)	41	148 (137,159)	135 (126,150)	4.0 (1.9,9.9)	0.99
	No	221	149 (134,160)	135 (128,147)	215	148 (133,160)	138 (132,150)	3.9 (1.7,6.1)	
baseline SBP reaching the targets	Yes	88	130 (110,135)	129 (122,144)	99	131 (109,136)	135 (128,149)	4.9 (1.1,8.6)	0.48
	No	173	155 (149,164)	136 (129,146)	157	158 (149,165)	139 (134,152)	3.0 (0.6,5.5)	

Data are showed as systolic blood pressure(mmHg):median, (IQR)

*BMI* body mass index, *SBP* systolic blood pressure, *IQR* interquartile range, *CI* confidence internal

### Secondary outcomes

#### BP control rate

Among patients not receiving antihypertensive treatment at baseline, 82 (31.4%) were in the intervention group and 79 (30.9%) were in the control group, with no significant difference between two groups (*P* > 0.05). For patients receiving treatment at baseline, the BP control rates were 34.6% and 37.3% in the intervention and control groups, respectively, with no significant difference (*P* > 0.05).

At the end of the follow-up period, the overall BP control rate increased by 36.4% in the intervention group and 22.3% in the control group from the baseline, with the intervention group showing a higher overall BP control rate (*P* < 0.05). The BP control rate in patients who did not receive antihypertensive treatment was 46.3% in the intervention group and 40.5% in the control group, with no significant difference (*P* > 0.05). However, in patients receiving therapy, the BP control rate increased by 33.5% in the intervention group and 14.1% in the control group from the baseline, with the intervention group showing a higher BP control rate (*P* < 0.05). Table 5.

**Table 5:** Blood pressure control rate before and after intervention in the two groups

	Intervention group (*n* = 261)				Control group (*n* = 256)				*P* values^a^
Participant	*n*	Baseline	Month 12	Change	*n*	Baseline	Month 12	Change	
Total	261	62 (23.6)	157 (60.2)	95 (36.4)	256	66 (25.8)	123 (48.1)	57 (22.3)	0.01
Patients not receiving therapy at baseline	82	0	38 (46.3)	38 (46.3)	79	0	32 (40.5)	34 (40.5)	0.46
Patients receiving therapy at baseline	179	62 (34.6)	119 (66.5)	60 (33.5)	177	66 (37.3)	91 (51.4)	25 (14.1)	＜0.01

Data are showed as numbers(percentages)

^a^Compared at the end of 12 months follow-up

#### Excessive BP reduction

In the intervention group, 22 patients (8.4%) had excessive BP reduction at baseline and 10 new-onset cases (3.8%) had excessive BP reduction during follow-up; however, all 32 patients were corrected at the end of follow-up. In the control group, 25 patients (9.4%) had excessive BP reduction at baseline, but 6 patients (1.2%) remained uncorrected. Additionally, 23 new-onset cases (9.0%) were found during follow-up, with five patients (2.0%) remaining uncorrected. The new-onset rate of excessive BP reduction was lower in the intervention group than that in the control group (3.8 vs 9.0%, *P* < 0.05).

In patients with frailty in the intervention group, six patients (15.0%) showed excessive BP reduction at baseline, with one new-onset case (2.5%) during follow-up, and all seven patients were finally corrected. In patients with frailty in the control group, seven patients (17.1%) showed excessive BP reduction at baseline, with three patients (7.3%) remaining uncorrected; there were eight new-onset cases (19.5%) with excessive BP reduction during follow-up, and three patients (7.3%) remained uncorrected. The new-onset rate of excessive BP reduction in patients with frailty was lower in the intervention group than that in the control group (2.5% vs. 19.5%, *P* < 0.05). Table 6.

**Table 6:** Excessive blood pressure reduction cases in the two groups

Participant	Group	n	Baseline	New-onset	Month 12		
					Uncorrected among baseline	Uncorrected among new-onsets	total
Total participants	intervention	261	22 (8.4)	10 (3.8)	0	0	0
	Control	256	25 (9.4)	23 (9.0)	3 (1.2)	5 (2.0)	8 (3.1)
*χ*^2^/ Fisher's Exact Probability Method			0.28	5.74	-	-	-
*P* values			0.60	0.02	0.12	0.03	<0.01
Participants with frailty	intervention	40	6 (15.0)	1 (2.5)	0	0	0
	Control	41	7 (17.1)	8 (19.5)	3 (7.3)	3 (7.3)	6 (14.6)
*χ*^2^/Fisher's Exact Probability Method			0.07	-			-
*P* values			0.80	0.03	0.24	0.24	0.03

Data are showed as numbers(percentages)

#### APP use in the intervention group

We extracted the number of home BP measurements and online consultations of 261 patients in the intervention group from Shanghai Zhizhong Medical Technology Co., Ltd. A total of 23,752 home BP measurements were uploaded, 24,445 online consultations were accumulated, and 361 remote adjustments for antihypertensive treatment were made. Among them, patients with frailty accumulated 4340 online consultations and 4293 home BP uploaded and received 104 online therapy adjustments.

Post-hoc analyses of online consultations and home BP measurement frequencies were conducted and stratified by age, education, and frailty. The results showed that patients aged median or above, with high school education or above, and with frailty had higher per capita numbers of online consultations and home BP measurements (*P* < 0.05). Supplementary eTable 1.

## Discussion

Previous researches have shown greater reductions in SBP and DBP for hypertensive patients used digital intervention during a 3-month follow-up; however, its long-term efficacy needs to be confirmed [25]. And mobile APP mostly were been used by younger patients, who are more proficient in using smartphones [24]. However, with the aging population, a growing number of older patients will gradually attempt this new model for BP management, which will be a trend in the future. Whether the older participants will gain benefit from digital intervention is unknown, this is the first study that evaluate the effectiveness of digital therapy for older hypertension patients.

In this study, we assessed the effectiveness of an internet-based platform-IHEC therapy model in older patients with hypertension. We found that the SBP of iHEC group was 4.2 mmHg lower than that of the control group after 12months follow-up, and the new-onset rate of excessive BP lowering in the iHEC group was lower than that in the control group. The effect size observed from this intervention could be expected to result in a reduction of 10% in patients having a stroke or coronary events and a reduction of 6% in patients having heart failure. Such effective model could make a major difference to millions of older hypertensive patients in China and worldwide.

When analyzing the results, there are several points worthy of attention: 1. For patients not receiving antihypertensive treatment at baseline, there was no significant difference in the BP control rates between the two groups (46.3% vs. 40.5%) at the end of follow-up. However, among those who received treatment, the intervention group had a significantly higher control rate than the control group (66.5% vs. 51.4%). This may be due to patients’ compliance, but the limited number of patients without treatment, and enlarging the sample size could be helpful in detecting differences. 2. Excessive BP reduction in the control group should not be overlooked, possibly because of misconceptions regarding the target of therapy, lack of awareness of the existence and dangers of excessive BP reduction, and irregular follow-up. The intervention group effectively avoided this problem with the help of the iHEC-APP and enhanced the detailed BP management. 3. Factors such as age, education level, and frailty affected the use of the iHEC-APP, but aging and frailty did not limit or obstruct the older from accepting intelligent management. These patients were even more dependent on this model. Older patients with frailty face more difficulties in maintaining their BP within a reasonable range, iHEC could provide a convenient and efficient platform for doctor-patient communication to achieve this goal. 4. Although the APP regularly reminded participants to upload lifestyle information such as medication, weight, exercise, diet, sleep, etc., patients were not active in collecting these data. It is necessary to optimize the APP and simplify the uploading process, such as providing smart bands for automatic import of exercise and sleep data, and reducing text input.

### Limitations

The study results should be considered in the context of the following limitations: 1. Lack of blinding: Because patients in the intervention group logged into the iHEC system with their real names, blinding was impossible for both participants and staff, and one pre-designated staff member was responsible for the enrollment of participants. However, allocation concealment before randomization was used to minimize the risk of bias. 2. Limited follow-up duration: This study was conducted during the COVID-19 pandemic. Pandemic and sporadic community lockdowns have hindered medical visits, possibly making patients more inclined to choose online treatment. Whether older patients would prefer the iHEC model for long-term BP management in the post-pandemic period requires an extended follow-up. 3.Uncertain generalizability: All participants came from communities in economically developed and well-educated cities with good network coverage and stable internet speeds. It is unknown whether these factors affect the effectiveness of this model when applied to other cities. However, the iHEC online platform requires only a smartphone application and internet for individuals, which has already widely penetrated in the general populations and thus poses minimum additional costs. Previous digital interventions for hypertension have proven to be cost effective. To properly assess the cost-effectiveness of iHEC model for older hypertensive patients in the long term, additional large-scale real-world study is needed, particularly in the low- to -middle income countries/setting, and we are in the process of such work.

### Perspective of Asia

Hypertension among Asian populations exhibited a stronger association with cardiovascular events such as stroke and myocardial infarction compared to Western populations. Despite this, the rate of achieving controlled blood pressure in most Asian countries and regions remains suboptimal [26]. Meanwhile, with the exacerbation of population aging, many Asian countries, especially those in East Asia, are forecast to become the most rapidly aging societies in the world. IHEC model is proved to be effective in the blood pressure management of older hypertensive patients, including people with frailty, that can serve as a reference for other aging societies.

## Conclusion

For older patients with hypertension, the digital iHEC therapy model achieved better SBP reduction and higher BP control rates, and reduced the risk of excessive BP reduction compared to those receiving conventional treatment alone. As a new choice for hypertension management, it is necessary to further optimize it in the future and evaluate its long-term effects and cost-effectiveness in large-scale studies.

## Novelty and relevance

### What is new?

- The iHEC is a new internet-based information platform that provides technical support for intelligent BP management. As an innovative model, iHEC overcomes the limitations of time and space, which are supposed to bring convenience to BP management in older patients. However, rigorous evaluation of its validity is lacking. Therefore, this study focused on the effectiveness of iHEC application through follow-up of 517 older patients with hypertension.

### What is relevant?

- With an increase in the aging population, the number of older patients with hypertension has been increasing. However, the current status of BP management in the older population remains unsatisfactory. Digital platforms have been integrated into the field of chronic disease therapy with the advantages of standardization, informatization, and intelligence. This study aimed to explore the effects of iHEC in older patients in order to provide evidence for its future clinical application.

### Clinical implications

- As an important supplement to conventional treatment, if a remote intelligent model based on iHEC can be confirmed to effectively improve BP management and solve the inconvenience of medical visits, it will bring good news to older patients and will be worthy of clinical promotion.

## Supplementary information

Supplementary eFigure 1

Supplementary Table 1
