# Supplementary material for: Effectiveness of the intelligent hypertension excellence centers (iHEC) therapy model in the blood pressure management of older hypertensive patients: a randomized controlled trial
Source: Hypertens Res. 2024 Nov 6;48(1):15–25. doi: 10.1038/s41440-024-01951-w (PMC11700847; doi:10.1038/s41440-024-01951-w)
Supplement: Supplementary file 1 — Supplementary eFigure 1 [file 41440_2024_1951_MOESM1_ESM.docx]

**SUPPLEMENT MATERIALS**


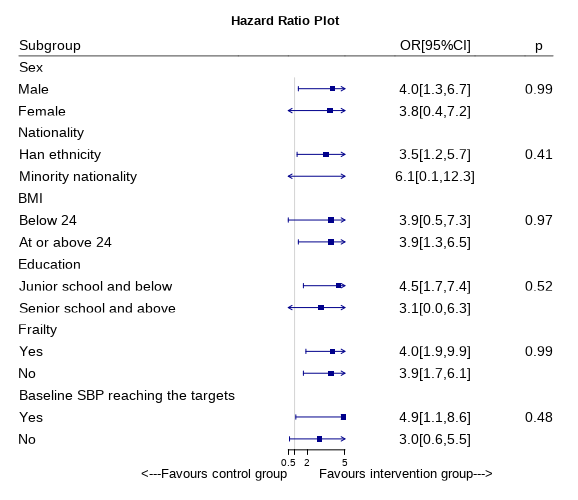


**Supplementary eFigure 1** Subgroup analyses showing effect sizes in intervention and control groups.
