# Supplementary material for: Effectiveness of the intelligent hypertension excellence centers (iHEC) therapy model in the blood pressure management of older hypertensive patients: a randomized controlled trial
Source: Hypertens Res. 2024 Nov 6;48(1):15–25. doi: 10.1038/s41440-024-01951-w (PMC11700847; doi:10.1038/s41440-024-01951-w)
Supplement: Supplementary file 2 — Supplementary Table 1 [file 41440_2024_1951_MOESM2_ESM.docx]

| **Supplementary Table 1** | | | | | | |
| --- | --- | --- | --- | --- | --- | --- |
| Item | | n | Online consultations | P  values ^a^ | Home BP measurements | P values ^a^ |
| Age  median,（IQR） | ＜median | 105 | 83(44,126) | 0.03 | 78(54,116) | 0.03 |
|  | ≥median | 156 | 98(67,134) |  | 93(75,118) |  |
| Education  median,（IQR） | Junior school and below | 136 | 83(51,118) | 0.02 | 81(61,112) | ＜0.01 |
|  | Senior school and above | 125 | 105(67,138) |  | 96(74,125) |  |
| Frailty  median,（IQR） | Yes | 40 | 110(77,138) | 0.04 | 104(85,132) | ＜0.01 |
|  | No | 221 | 93(57,127) |  | 86(61,112) |  |
|  | | | | | | |
